# Dietary ε-Polylysine Affects on Gut Microbiota and Plasma Metabolites Profiling in Mice

**DOI:** 10.3389/fnut.2022.842686

**Published:** 2022-04-28

**Authors:** Xuelei Zhang, Baoyang Xu, Zhenping Hou, Chunlin Xie, Yaorong Niu, Qiuzhong Dai, Xianghua Yan, Duanqin Wu

**Affiliations:** ^1^Institute of Bast Fiber Crops, Chinese Academy of Agricultural Sciences, Changsha, China; ^2^Department of Animal Nutrition and Feed Science, College of Animal Sciences and Technology, Huazhong Agricultural University, Wuhan, China

**Keywords:** ε-polylysine, glycerophospholipid metabolism, gut microbiota, growth performance, C57 mice

## Abstract

Given the antibacterial effects of ε-polylysine acting on cell membranes, and that glycerol phospholipids are important components of the cell membrane, we hypothesized that ε-polylysine may regulate glycerophospholipid metabolism by modifying the gut microbiota. To test this hypothesis, we treated post-weaning C57 mice with different levels of ε-polylysine (0, 300, 600, and 1,200 ppm) in their basic diet. The growth performance and morphology of intestine were then determined. Modification of the gut microbiota and their function were analyzed using 16S rDNA sequencing. Metabolite identification was performed using the LC-MS method. The results showed that body weight decreased with an increasing supplemental level of ε-polylysine from 5 to 7 weeks (*P* < 0.05), but no significant difference was observed after 8 weeks (*P* > 0.05). Supplementation with 1,200 ppm ε-polylysine changed the morphology of the jejunum and ileum, increased the villus length, decreased the crypt depth of the jejunum, and decreased the villus length and crypt depth of the ileum (*P* < 0.05). ε-Polylysine shifted the intestine microbiota by changing alpha diversity (Chao 1, observed species, Shannon, and Simpson indices) and varied at different times. ε-polylysine decreased Firmicutes and increased Bacteroidetes at 4 week, but increased Firmicutes and decreased Bacteroidetes at 10 week. ε-Polylysine regulated genera associated with lipid metabolism such as *Parabacteroides*, *Odoribacter*, *Akkermansia*, *Alistipes*, *Lachnospiraceae* UCG-001, *Collinsella*, *Ruminococcaceae*, and *Intestinimonas*. During the adult period, the genera *Alistipes*, *Lachnospiraceae* UCG-001, and *Streptomyces* were positively associated with PC, PE, LysoPC, LysoPE, 1-Arachidonoylglycerophosphoinositol and OHOHA-PS (*R* > 0.6, *P* < 0.001), but changes in *Blautia*, *Christensenellaceae* R-7 *group*, *Odoribacter*, *Allobaculum*, *Ruminococcaceae* UCG-004, *Ruminococcaceae* UCG-005, and *Lachnospiraceae* UCG-010 were negatively correlated with glycerophospholipid metabolites (*R* < −0.6, *P* < 0.001). The abundance of glycerophospholipid metabolites, including PC, PE, lysoPC, and lysoPE, were decreased by ε-polylysine. Furthermore, ε-polylysine reduced the incidence of the genera including *Ruminococcus*, *Prevotella*, *Prevotellaceae*, *Butyricimonas*, and *Escherichia-Shigella* and reduced the abundance of *Faecalibaculum*, *Christensenellaceae* R-7 *group*, *Coriobacteriaceae* UCG-002. In conclusion, ε-polylysine modified gut microbiota composition and function while also restraining pathogenic bacteria. The glycerophospholipid metabolism pathway and associated metabolites may be regulated by intestinal bacteria.

## Introduction

It is important to understand the relationship between the gut microbial ecosystem and host lipid homeostasis. The gut microbiota significantly influences the body’s lipidome (1). Host lipid homeostasis and the gut microbial ecosystem are closely associated with metabolic diseases (2). Phospholipids are important components of all cellular and subcellular membranes (3), and are involved in the pathogenesis of several diseases, including type 2 diabetes, tuberculosis (4), fatty liver disease (5), and schizophrenia-related behaviors (6). Glycerophospholipids includes phosphatidylcholine (PC) and phosphatidylethanolamine (PE), which are components of plasma lipoproteins that transport neutral lipids (3). Different types of phospholipids have unique physiological functions. PC is the major membrane-forming phospholipid in eukaryotes (7). In the livers of PC-treated rats, PC restored intestinal barrier function and reduced endotoxemia, demonstrating its hepatoprotective properties (8). The gut microbiota can detect free cholesterol and elevate levels of PC and PE in specific pathogen-free and germ-free mice (9). Microbes can significantly alter glycerophospholipid acyl chain profiles and enhance the levels of glycerophospholipid precursors (10).

ε-Polylysine, a homopolymer, composed of 25–35 mono-lysine, which connected by amide linkages between ε-amino and α-carboxyl groups (11). Interestingly, ε-polylysine exhibits a broad spectrum of bacteriostatic properties that affect gram-positive bacteria, such as *Listeria monocytogenes*, *Escherichia coli* O157:H7, *Salmonella typhimurium*, *Staphylococcus aureus*, and *Bacillus subtilis* (12–15). The bacteriostatic properties of ε-polylysine was in a highly polymerized multivalent cationic state, and its antibacterial activity mechanism stems from the fact that the surface of bacteria is an anion that provides potential sites for electrostatic binding of ε-polylysine cationic surfactants (16, 17). Supplementing mice diets with antimicrobial ε-polylysine altered the gut microbial community in mice and their respective predicted functions, suggesting that the microbiome is dynamic, resilient, and adapts to microbially active dietary components (18). In our previous study based on pigs, we found that ε-polylysine may regulate the gut microbiota and its function, as it influences the utilization of lipids and metabolizable energy (19). Furthermore, ε-polylysine decreased serum and liver lipid content, and enhanced the activity of two key enzymes named hepatic acetyl-coenzyme A carboxylase and glucose-6-phosphate dehydrogenase, which involved in fatty acid biosynthesis in rats (20). ε-polylysine also inhibits pancreatic lipid activity in rats by suppressing dietary fat absorption from the small intestine (21).

Owing to the effect of ε-polylysine on microorganisms and phospholipid metabolism, we aimed to establish a comprehensive overview of microbial colonization affecting plasma glycerophospholipid metabolism in C57 mice. In this study, we performed 16S rDNA analysis and metabolome analysis to systematically investigate the influence of the food additive ε-polylysine on the intestinal microflora and glycerophospholipid metabolism in C57 mice. We aimed to provide important data for the development and application of food additives to improve the safety of human food products.

## Materials and Methods

### Experiments Design, Specimen Collection

Our animal experimental procedures were approved by the Institute of Bast Fiber Crops, CAAS (Changsha, China), and Huazhong Agricultural University (Wuhan, China). All experiments in this study were conducted with the Scientific Ethics Committee of the Huazhong Agricultural University in strict accordance (approval number: HZAUMO-2019-059). Three-week-old post-weaning specific pathogen-free C57 Black mice (*n* = 80, body weight 9.35 ± 1.13 g) were randomly assigned to four groups, each containing 20 male mice. Each group was fed the same diet (AIN-76A) supplemented with 0, 300, 600, and 1,200 ppm ε-polylysine. C57 mice were maintained under 12:12 h light/dark conditions at Huazhong Agricultural University with free access to feed and water. Body weights were measured at the beginning of the experimental period, and then once per week. Fecal samples (*n* = 12 each group) were collected at weeks four, six, and ten. At the end of the experiment (after about 10 weeks), the mice were anesthetized and sacrificed, and intestine and plasma samples (*n* = 20 each group) were harvested.

### Histopathology Examination of Intestine Tissue

The morphology of the intestine was determined using hematoxylin and eosin. One centimeter of the small intestine and large intestine, as well as the middle position of each bowel segment, were rinsed in PBS, fixed in formalin overnight at 4°C, and embedded in paraffin. Intestinal sections were stained with H&E. Villus height and crypt depth of the small intestine were measured using Image-Pro Plus 6.0 software (Media Cybernetics, Inc., Rockville, MD, United States).

### Intestinal Microbiota Analysis

Qubit dsDNA Assay Kit (Cat. No. Q328520, Life Technologies, Carlsbad, CA, United States) was used to extract DNA according to the manufacturer’s instructions. The diluted DNA (1 ng/μL) was used as a template for polymerase chain reaction (PCR) amplification of bacterial 16S rDNA genes. For bacterial diversity analysis, the V3–V4 variable regions of 16S rDNA genes were amplified using the universal primers 343F (5′-TACGGRAGGCAGCAG-3′) and 798R (5′-AGGGTATCTAATCCT-3′) (22). The final amplicon was quantified using the Qubit dsDNA Assay Kit (Life Technologies, Q328520, Grand Island, CA, United States). Equal amounts of purified amplicons were pooled for sequencing on the Illumina MiSeq platform.

Clustering to generate OTUs using Vsearch software (23) with a 97% similarity cutoff. The representative reads of each OTU were selected using the QIIME package (version 1.8.0). All representative reads were annotated and blasted against the Silva database Version 123 (or Greengens) using the RDP classifier (24) with a confidence threshold of 70%.

The abundance of each OTU was normalized relative to the sample with the fewest sequences. Alpha diversity was used to analyze the complexity of species in fecal sample by applying the Chao1, observed species, Shannon, and Simpson indices. Beta diversity was used to evaluate the complexity of species and was calculated using PCoA. Both Alpha and Beta diversity were used in the QIIME software (version 1.8.0). The different phyla and genera of 16S rDNA were analyzed using the Kruskal-Wallis test. *P* < 0.01 was considered highly significant, and *P* < 0.05, was considered significant. We confirmed that all species were differentially abundant by LEfSe analysis. Heatmap diagrams and other plots were created in the R environment (v3.1.2). Functional profiles of the gut microbiota were analyzed using PICRUSt (25). OTUs were normalized, and the gene categories were at level 2 of the KEGG orthology groups (26).

### Metabolomics Analysis of Plasma

Plasma samples (100 μL) were added into 10 μL internal standard (2-chloro-l-phenylalanine in methanol, 0.3 mg/mL) dissolved in methanol, and vortexed for 10 s. Then, the mixtures were vortexed for 1 min with 300 μL mixture of methanol and acetonitrile (2/1, v/v). All samples were ultrasonicated at 25–28°C for 10 min and stored at −20°C for 30 min. The extract was centrifuged at 13,000 rpm at 4°C for 15 min. The supernatant (300 mL) was dried in a freeze-concentration centrifugal dryer. A mixture of 400 μL methanol and water (1/4, vol/vol) was added to each sample, then vortexed for 30 s, and placed at 4°C for 2 min. Samples were centrifuged at 13,000 rpm at 4°C for 5 min. The supernatant (150 μL) from each tube was collected using crystal syringes, filtered through 0.22 μm microfilters and transferred to LC vials. The vials were stored at −80°C until LC -MS analysis. The analysis instrument of metabolic profiling was the AB ExionLc system (AB SCIEX, Framingham, MA, United States) coupled with an AB SCIEX Triple TOF 6600 System (AB SCIEX, Framingham, MA, United States). An ACQUITY UPLC BEH C18 column (1.7 μm, 2.1 × 100 mm) were employed in both positive and negative modes. The binary gradient elution system consisted of (A) water (containing 0.1% formic acid, v/v) and (B) acetonitrile (containing 0.1% formic acid, v/v) and separation was achieved using the following gradient: 0 min, 95% A, 5% B; 2 min, 80% A, 20% B; 4 min, 75% A, 25% B; 9 min, 40% A, 60% B; 14 min, 100% B; 16 min, 100% B; 16.1 min, 95% A, 5% B and 18.1 min, 95% A, 5% B. The flow rate was 0.4 mL/min and column temperature was 45°C. All the samples were kept at 4°C during the analysis. Data acquisition was performed in full scan mode (m/z ranges from 70 to 1,000) combined with IDA mode. Parameters of mass spectrometry were as follows: ion source temperature, 550°C (+) and 550°C (−); ion spray voltage, 5,500 V (+) and 4,500 V (−); curtain gas of 35 PSI; declustering potential, 80 V (+) and −80 V (−); collision energy, 10 eV (+) and −10 eV (−); and interface heater temperature, 550°C (+) and 550°C (−). For IDA analysis, range of m/z was set as 50–1,000, the collision energy was 30 eV.

The acquired LC-MS raw data were analyzed using Progenesis QI software (Waters Corporation, Milford, CT, United States). Metabolites were identified using Progenesis QI (Waters Corporation, Milford, CT, United States) data processing software, based on public databases such as http://www.hmdb.ca/, http://www.lipidmaps.org/, and self-built databases. PCA and OPLS-DA were carried out to visualize the metabolic alterations. Hotelling’s T2 region, shown as an ellipse in the score plots of the models, defined the 95% confidence interval of the modeled variation. Variable importance in the projection (VIP) ranks the overall contribution of each variable to the OPLS-DA model, and variables with VIP > 1 were considered relevant for group discrimination. The differential metabolites were selected on the basis of the combination of a statistically significant threshold of variable influence on projection (VIP) values obtained from the OPLS-DA model and *p* values from a two-tailed Student’s t-test on the normalized peak areas, where metabolites with VIP values larger than 1.0, and *p* values less than 0.05, were considered as differential metabolites (27).

### Correlation Coefficient Analyses

Spearman’s rank correlation coefficient analysis was performed to examine the possible connection between the composition of intestinal microflora at the genus level and plasma metabolites in C57 mice.

## Results

### Effects of Dietary Interventions on Body Weight and Intestine Morphology

**FIGURE 1:**
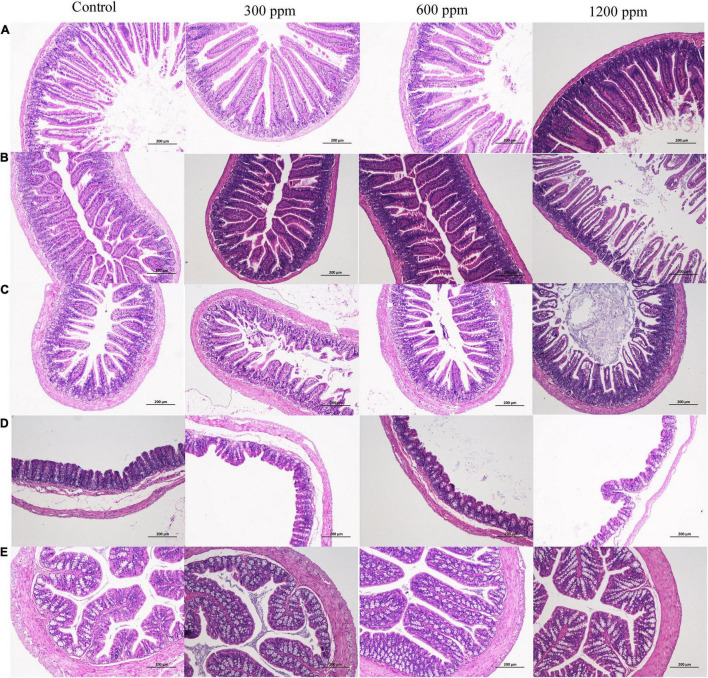
Microscopic observation of intestine. **(A)** H&E (100×) staining of crypts and villi on duodenum between four groups; **(B)** H&E (100×) staining of crypts and villi on jejunum between four groups; **(C)** H&E (100×) staining of crypts and villi on ileum between four groups; **(D)** H&E (100×) staining of crypts and villi on cecum between four groups; **(E)** H&E (100×) staining of crypts and villi on colon between four groups.

The effects of dietary supplementation with ε-polylysine on body weight were shown in Table 1. At the beginning of the experiment, there were no significant differences in body weight (*P* > 0.05), while the weights in the control group and 300 ppm group were significantly higher than those in the 1,200 ppm group from 5 to 7 weeks (*P* < 0.05). After 8 week, the weight of mice did not differ between the four groups (*P* > 0.05), but the trend of weight decreased with increasing ε-polylysine supplementation. As shown in Table 2, there was no significant difference in villus length and crypt depth in the duodenum (*P* > 0.05). The villus length in the 1,200 ppm group was significantly higher than that in the other groups (*P* < 0.05), but the crypt depth in the 300 and 1,200 ppm groups were significantly lower than that in the control group (*P* < 0.05). The villus length in the 1,200 ppm group was significantly lower than that in 600 ppm group, and crypt depth in the 1,200 ppm group was significantly lower than that in the control group (*P* < 0.05). Compared with the control group, damage to the jejunum epithelium was observed in the 1,200 ppm group (Figure 1).

**TABLE 1:** The changes of C57 mice weight during the experimental period.

Item/g	Groups				*P*-Value	95% CI
	Control	300 ppm	600 ppm	1,200 ppm		
3 weeks	9.369	9.401	9.289	9.358	0.9925	9.093–9.615
4 weeks	15.48	16.36	15.68	15.23	0.3639	15.211–16.112
5 weeks	20.34^a^	20.69^a^	20.17^ab^	18.72^b^	**0.0040**	19.500–20.366
6 weeks	22.41^a^	22.61^a^	22.04^ab^	20.52^b^	**0.0042**	21.347–22.301
7 weeks	23.57^a^	24.19^a^	23.43^ab^	21.87^b^	**0.0031**	22.674–23.682
8 weeks	24.61	24.38	24.60	23.05	0.1037	23.603–24.663
9 weeks	25.69	25.68	25.47	24.47	0.1913	24.851–25.777
10 weeks	26.27	26.40	26.44	25.45	0.4020	25.653–26.608

*Data are expressed as MEAN with 95% CI; Means with different letters within a column differ (P < 0.05); n = 20 per treatment. Treatment Control group, 0 ppm ε-polylysine; experimental groups: 300, 600, and 1,200 ppm ε-polylysine. Arrange the averages from largest to smallest, and mark the letter “a” after the largest average. Use the mean to compare with each mean in turn, and mark the same letter “a” for the insignificant difference until the mean with significant difference is encountered, then mark the letter “b”. P < 0.05, significantly different.*

**TABLE 2:** The villus length and crypt depth of small intestine.

Item/μm	Groups				*P*-value	95% CI
	Control	300 ppm	600 ppm	1,200 ppm		
Villus length of duodenum	447.6	442.9	452.3	431.2	0.6328	429.867–455.244
Crypt depth of duodenum	95.20	92.00	91.04	87.42	0.2658	88.391–94.009
Villus length of jejunum	253.5^b^	252.4^b^	267.5^b^	311.8^a^	**<0.0001**	259.754–278.546
Crypt depth of jejunum	100.1^a^	82.27^b^	90.00^ab^	84.20^b^	**0.0009**	85.681–91.999
Villus length of ileum	185.4^ab^	169.6^ab^	195.5^a^	169.9^b^	**0.0309**	172.235–186.595
Crypt depth of ileum	108.1^a^	97.85^ab^	103.8^ab^	93.53^b^	**0.0228**	96.622–104.241

*Data are expressed as MEAN with 95% CI; Means with different letters within a column differ (P < 0.05); n = 6 per treatment. Treatment Control group, 0 ppm ε-polylysine; experimental groups: 300, 600, and 1,200 ppm ε-polylysine. Arrange the averages from largest to smallest, and mark the letter “a” after the largest average. Use the mean to compare with each mean in turn, and mark the same letter “a” for the insignificant difference until the mean with significant difference is encountered, then mark the letter “b”. P < 0.05, significantly different.*

### Effects of Dietary Interventions on Gut Microbiota on 4 Week-C57 Mice

**FIGURE 2:**
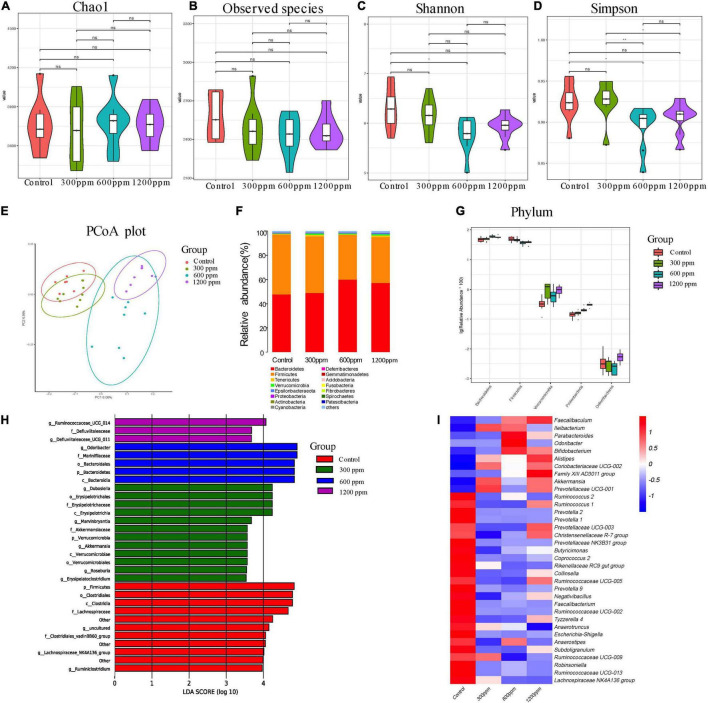
Shifts in the fecal bacterial composition of C57 mice at 4 weeks. **(A)** Bacterial alpha diversity determined by Chao1 index. **(B)** Bacterial alpha diversity determined by Observed species index. **(C)** Bacterial alpha diversity determined by Shannon index. **(D)** Bacterial alpha diversity determined by Simpson index. **(E)** PCoA was performed at the operational taxonomic unit (OTU) level based on Bray–Curtis metrics for all samples at different time. **(F)** Comparison of the relative abundance of top 15 phyla. **(G)** The different phylum compositions of the experimental group. **(H)** LefSe results from the fecal microbiota indicating genera significantly associated with the control and ε-polylysine groups samples. **(I)** Heat map and hierarchical clustering of differentially abundant gut bacterial at genus level.

Among the bioinformatic analyses, a total of 77,742, 77,686, 77,120, and 75,051 valid tags were obtained for the four groups, accounting for 90.62% (control group), 91.40% (300 ppm group), 91.93% (600 ppm group), and 91.22% (1200 ppm group) of raw sequences, respectively (Supplementary Data 1). Taxonomic analysis revealed a total of 17 bacterial phyla, 35 classes, 93 orders, 149 families, 319 genera, and 88 species. As shown in Figures 2A–D, there were no significant differences in the alpha diversity indices (Chao1, observed species, and Shannon indices) among the four groups (*P* > 0.05), but 600 ppm group had a lower Simpson index than the control group and 300 ppm group (*P* < 0.05), while the Simpson index at 300 ppm was significantly different from that in the 1,200 ppm group (*P* < 0.05; Figure 2D). The microbial community structure of all samples (beta diversity) was visualized using PCoA (Figure 2E). The two factors (PC1 and PC2) accounted for 8.08 and 6.36% of the sample variation, respectively. The PCoA based on the bacterial OTUs showed that the samples clustered together, which indicated a shift in the gut bacterial community among the four groups (Figure 2E). Our results showed that the abundance of phylum Bacteroidetes was significantly increased by ε-polylysine, and the abundance of phylum Firmicutes was significantly decreased (*P* < 0.05), which was a domain phylum in all samples (Figures 2F,G and Supplementary Data 2). The abundance of Proteobacteria and Verrucomicrobia was higher in the ε-polylysine groups than in the control group (*P* < 0.05; Figure 2G). A total of 96 different genera were identified by Wilcoxon text analysis (Supplementary Data 3). The LEfSe analysis revealed that the genera had effects among the four groups (Figure 2H). Ten genera were more abundant in the ε-polylysine group, including *Faecalibaculum*, *Ileibacterium*, *Parabacteroides, Odoribacter, Bifidobacterium, Alistipes, Coriobacteriaceae* UCG-002, *Family* XIII AD3011 *group, Akkermansia*, and *Prevotellaceae* UCG-001.

The others were more abundant in the control group, including *Ruminococcus*, *Prevotella*, *Prevotellaceae* UCG-003, *Christensenellaceae* R-7 *group*, *Prevotellaceae* NK3B31 *group*, *Butyricimonas*, *Coprococcus* 2, *Rikenellaceae* RC9 *gut group*, *Collinsella*, *Ruminococcaceae*, *Negativibacillus*, *Faecalibacterium*, *Tyzzerella* 4, *Anaerotruncus*, *Escherichia-Shigella*, *Anaerostipes*, *Subdoligranulum*, *Robinsoniella*, and *Lachnospiraceae* NK4A136 *group* (Figure 2I and Supplementary Data 3).

### Effects of Dietary Interventions on Gut Microbiota on 6 Week-C57 Mice

**FIGURE 3:**
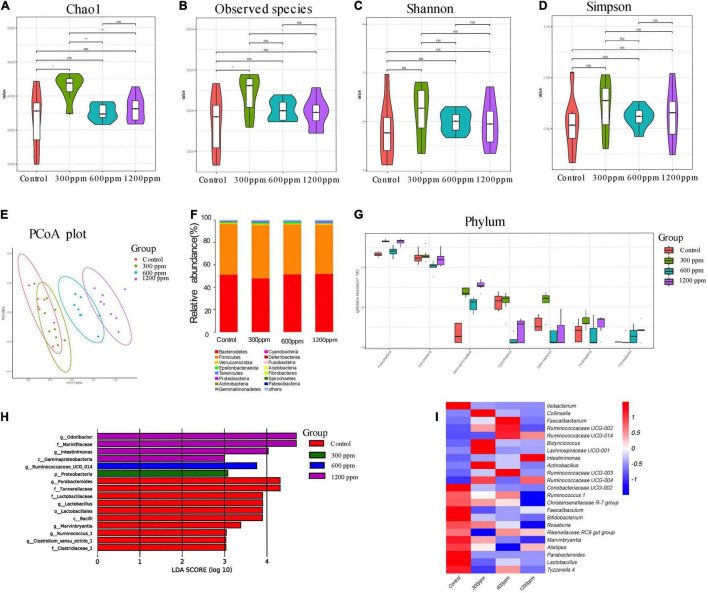
Shifts in the fecal bacterial composition of C57 mice at 6 weeks. **(A)** Bacterial alpha diversity determined by Chao1 index. **(B)** Bacterial alpha diversity determined by Observed species index. **(C)** Bacterial alpha diversity determined by Shannon index. **(D)** Bacterial alpha diversity determined by Simpson index. **(E)** PCoA was performed at the operational taxonomic unit (OTU) level based on Bray–Curtis metrics for all samples at different time. **(F)** Comparison of the relative abundance of top 15 phyla. **(G)** The different phylum compositions of the experimental group. **(H)** LefSe results from the fecal microbiota indicating genera significantly associated with the control and ε-polylysine group samples. **(I)** Heat map and hierarchical clustering of differentially abundant gut bacterial at genus level.

For the four groups, 76,009, 78,823, 78,996, and 77,816 valid tags were obtained, which accounted for 92.03, 91.92, 91.70, and 92.30% of the raw sequences, respectively (Supplementary Data 1). We examined the taxonomic composition of the fecal microbiota in 6 week-C57 mice, a total of 17 bacterial phyla, 37 classes, 104 orders, 183 families, 381 genera, and 99 species. The Chao1 index in 300 ppm group was significantly higher than that in the other groups (*P* < 0.05). The observed species index was significantly higher than that in the control group and the 1,200 ppm group (*P* < 0.05; Figures 3A–D). In the PCoA analysis, PC1 accounted for 7.82% and PC2 accounted for 6.96% (Figure 3E). Bacteroidetes and Firmicutes were the domain phyla, which is the same to 4 weeks (Figure 3F). There were seven different phyla among the four groups (Figure 3G and Supplementary Data 4). The abundance of the phyla Gemmatimonadetes, Proteobacteria, and Fibrobacteres was up-regulated by ε-polylysine. After Wilcoxon text analysis, we identified 140 different genera (Supplementary Data 5). In the control group, the genera *Clostridium sensu strcto* 1, *Ruminococcus* 1, *Marvinbryantia*, *Lactobacillus*, and *Parabacteroides* played an important role in LEfSe analysis. The genera *Ruminococcaceae* UCG 014, *Odoribacter*, and *Intestinimonas* affected the ε-polylysine groups (Figure 3H). Ten genera were upregulated, including *Collinsella*, *Faecalibacterium, Ruminococcaceae, Butyricicoccus, Lachnospiraceae* UCG-001, *Intestinimonas*, and *Actinobacillus*, while the other 13 genera were down-regulated, including *Ileibacterium*, *Coriobacteriaceae* UCG-002, *Ruminococcus* 1, *Christensenellaceae* R-7 *group*, *Faecalibaculum*, *Bifidobacterium*, *Roseburia*, *Rikenellaceae* RC9 *gut group*, *Marvinbryantia*, *Alistipes*, *Parabacteroides*, *Lactobacillus*, and *Tyzzerella* 4 (Figure 3I and Supplementary Data 5).

### Effects of Dietary Interventions on Gut Microbiota on 10 Week-C57 Mice

**FIGURE 4:**
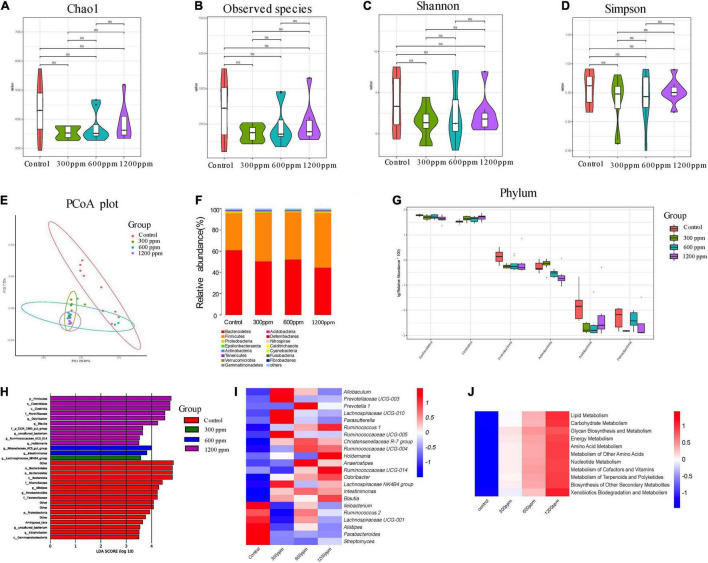
Shifts in the fecal bacterial composition of C57 mice at 10 weeks. **(A)** Bacterial alpha diversity determined by Chao1 index. **(B)** Bacterial alpha diversity determined by Observed species index. **(C)** Bacterial alpha diversity determined by Shannon index. **(D)** Bacterial alpha diversity determined by Simpson index. **(E)** PCoA was performed at the operational taxonomic unit (OTU) level based on Bray–Curtis metrics for all samples at different time. **(F)** Comparison of the relative abundance of top 15 phyla. **(G)** The different phylum compositions of the experimental group. **(H)** LefSe results from the fecal microbiota indicating genera significantly associated with the control and ε-polylysine group samples. **(I)** Heat map and hierarchical clustering of differentially abundant gut bacterial at genus level. **(J)** Heat map of KEGG pathway by PICRUSt analysis.

We found 59,729, 67,909, 70,719, and 74,870 valid tags for the four groups, which accounted for 79.89, 89.97, 84.83, and 87.36% of raw sequences, respectively (Supplementary Data 1). Taxonomic analysis revealed a total of 23 phyla, 54 classes, 132 orders, 225 families, 417 genera, and 127 species. As shown in Figure 4, the alpha diversity, including Chao1 index, observed species index, Shannon index, and Simpson index did not differ between the groups (Figures 4A–D). The PC1 accounted for 20.81% and PC2 accounted for 7.72%, as observed using PCoA analysis (Figure 4E). The abundance of the phylum Bacteroidetes was significantly decreased by ε-polylysine, and the abundance of phylum Firmicutes was significantly increased by ε-polylysine (*P* < 0.05), which was opposite to those at 4 weeks (Figure 4F). In addition, we identified six different phyla among the groups, and the abundance of Proteobacteria, Bacteroidetes, and Patescibacteria were down-regulated by ε-polylysine (Figure 4G and Supplementary Data 6). Sixty-four different genera were identified by Wilcoxon text analysis (Supplementary Data 7). After LEfSe analysis, the genera *Alistipes*, *Parabacteroide*s, and *Edaphobacter* played an important role in the control group, and the genus *Lachnospiraceae* NK4B4 *group* had an effect in 300 ppm group (Figure 4H). In addition, the *Rikenellaceae* RC9 *gut group* and *Intestinimonas* had effects at 600 ppm, and the genera *Odoribacter*, *Blautia*, *Ruminococcaceae* UCG-014, and *Holdemania* had effects in the 1,200 ppm group (Figure 4H). Furthermore, the results showed several upregulated genera, including *Allobaculum*, *Prevotellaceae* UCG-003, *Prevotella* 1, *Lachnospiraceae* UCG-010, *Parasutterella*, *Ruminococcus* 1, *Ruminococcaceae*, *Christensenellaceae* R-7 *group*, *Holdemania*, *Anaerostipes*, *Odoribacter*, *Lachnospiraceae* NK4B4 *group*, *Intestinimonas and Blautia*, and six down-regulated genera, including *Ileibacterium*, *Ruminococcus* 2, *Lachnospiraceae* UCG-001, *Alistipes*, *Parabacteroides*, and *Streptomyces* (Figure 4I and Supplementary Data 7). We investigated the functional profiles of microbiota by PICRUSt analysis, the relative abundances of genes involved in the metabolism of lipids, carbohydrates, glycans, energy, amino acids, other amino acids, cofactors and vitamins, terpenoids and polyketides, and nucleotides (Figure 4J and Supplementary Data 8). Additionally, the results showed increases in the proportions of genera involved in genetic information processing and cellular processes (Supplementary Data 8).

### Effects of Dietary Interventions on Blood Metabolomic Profiles

**FIGURE 5:**
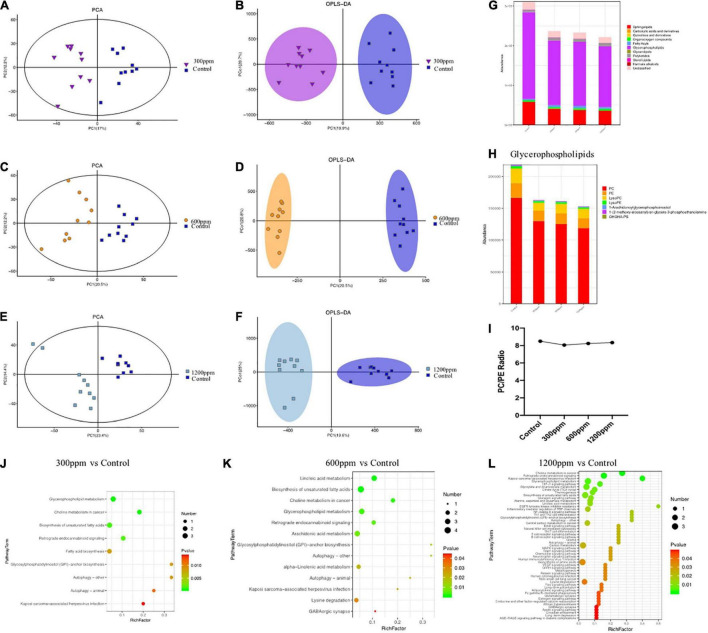
Metabolic patterns in the experiment groups. **(A)** PCA analysis between control group and 300 ppm group. **(B)** Clustering analysis of OPLS-DA in the control group and 300 ppm group. **(C)** PCA analysis between control group and 600 ppm group. **(D)** Clustering analysis of OPLS-DA in the control group and 600 ppm group. **(E)** PCA analysis between control group and 600 ppm group. **(F)** Clustering analysis of OPLS-DA in the control group and 600 ppm group. **(G)** Comparison of the abundance of major metabolite classifications. **(H)** Comparison of the abundance of Glycerophospholipids. **(I)** PC/PE radio. **(J)** The pathways in the control group and 300 ppm group. **(K)** The pathways in the control group and 600 ppm group. **(L)** The pathways in the control group and 1,200 ppm group.

**FIGURE 6:**
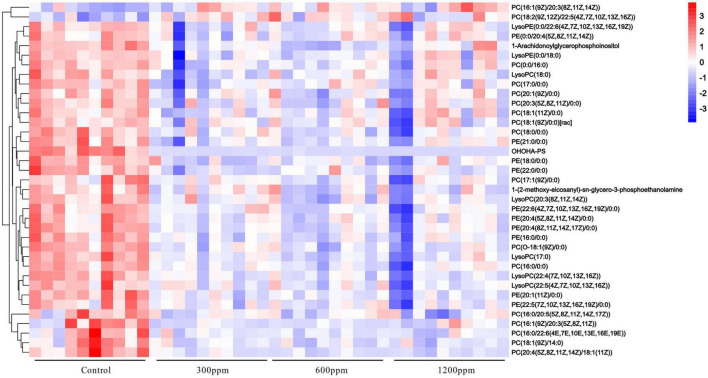
The heatmap of different metabolites.

The metabolomics data of the plasma were shown in Figure 5. In 300 ppm group compared with the control group, PC1 accounted for 17% and PC2 accounted for 12.2% (Figure 5A). The PC1 accounted for 20.5% and the PC2 accounted for 12.2%, between 600 ppm and the control group (Figure 5C). PC1 accounted for 23.4%, and PC2 accounted for 14.4% compared to the 1,200 ppm group compared to the control group (Figure 5E). The results of orthogonal partial least-squares discriminant analysis (OPLS-DA) of the liver between the ε-polylysine groups and the control group were presented in Figures 5B,D,F. The abundance of glycerophospholipids was the highest among all the lipid subclasses (Figure 5G). The abundance of organooxygen compounds, fatty acids, and polyketide subclasses were increased by ε-polylysine, but other metabolites such as sphingolipids and glycerophospholipids were decreased by ε-polylysine (Figure 5G). We further examined the taxonomic composition of the glycerophospholipid metabolites and found that PC, PE, and LysoPC were domain metabolites among all groups. The abundance of PC, PE, lysoPC, LysoPE, 1-Arachidonoylglycerophosphoinositol, 1-(2-methoxy-eicosanyl)-sn-glycero-3-phosphoethanolamine, and OHOHA-PS were decreased by ε-polylysine (Figure 5H and Supplementary Data 9). The PC/PE radio of control group and ε-polylysine had no significant changes (Figure 5I). The different metabolites detail was shown in the Figure 6. Plasma metabolites were annotated and were highly enriched in the pathways belonging to “glycerophospholipids metabolism” and “biosynthesis of unsaturated fatty acids” compared to the control groups (Figures 5J–L).

### Associations Between Fecal Microbiome and Metabolites in Plasma

**FIGURE 7:**
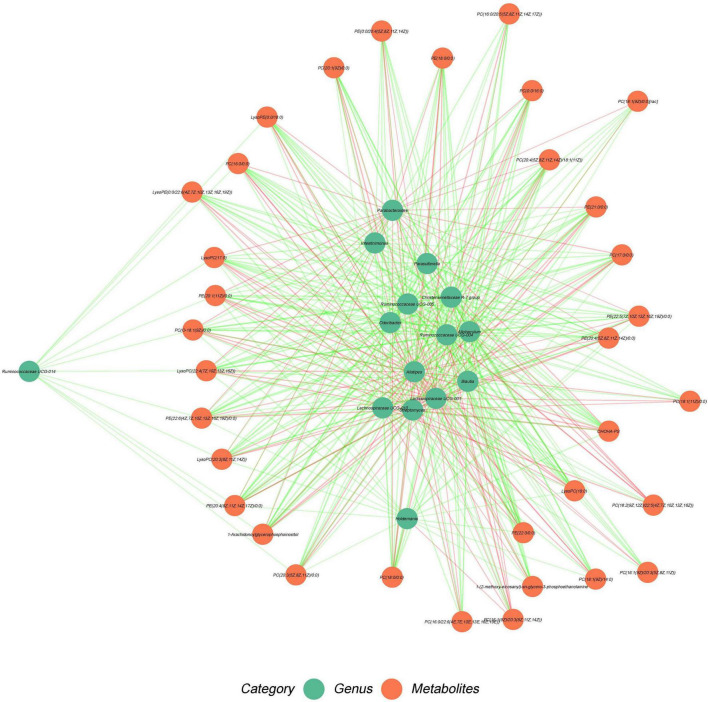
Association of fecal microbiome with liver metabolites. Green nodes represent metabolites and red nodes represent microbial genera. Red edges indicate positive correlations between metabolites and microbial genera. Spearman’s rank correlation coefficient > 0.5, *P* < 0.05. Green edges indicate negative correlation between metabolites and microbial genera. Spearman’s rank correlation coefficient < –0.5, *P* < 0.05.

We explored the association between fecal microbiota and the different metabolites shared by the plasma. Spearman correlation analysis showed that the metabolites containing PC(18:0/0:0), PC(0:0/16:0), PC[O-18:1(9Z)/0:0], PC[20:1(9Z)/0:0], LysoPC(18:0), and LysoPC[22:4(7Z,10Z,13Z,16Z)] were positively correlated with *Alistipes* (*R* > 0.6, *P* < 0.001), and PC(18:0/0:0), PC(17:0/0:0), LysoPC(18:0) were positively associated with *Lachnospiraceae* UCG-001 (*R* > 0.6, *P* < 0.001). In addition, PC[20:3(5Z,8Z,11Z)/0:0] was positively correlated with *Streptomyce*s (*R* > 0.6, *P* < 0.001). The metabolites PC(18:0/0:0), PC(16:0/0:0), PC(0:0/16:0), PC[20:3(5Z,8Z,11Z)/0:0], PC(17:0/0:0), PC[O-18:1(9Z)/0:0], PC[20:1(9Z)/0:0], PC[20:4(5Z,8Z,11Z,14Z)/18:1(11Z)], LysoPC(18:0), LysoPC[20:3(8Z,11Z,14Z)], lysoPC [22:4(7Z,10Z,13Z,16Z)], and lysoPC (17:0) were negatively associated with *Christensenellaceae* R-7 *group* and *Allobaculum*, respectively (*R* < −0.6, *P* < 0.001). The metabolite PC(18:0/0:0), PC[O-18:1(9Z)/0:0], PC[20:1(9Z)/0:0], and LysoPC(18:0) were negatively correlated with changes in *Blautia* (*R* < −0.6, *P* < 0.001), and PC(16:0/0:0), PC[20:4(5Z,8Z,11Z,14Z)/18:1(11Z)], LysoPC(17:0), and LysoPC[22:4(7Z,10Z,13Z,16Z)] were negatively correlated with *Ruminococcaceae* UCG-004 (*R* < −0.6, *P* < 0.001). PC(O-18:1(9Z)/0:0), LysoPC(17:0), and LysoPC[22:4(7Z,10Z,13Z,16Z)] were negatively associated with changes in *Odoribacter* (*R* < −0.6, *P* < 0.001; Figure 7 and Supplementary Data 10).

The metabolites PE[20:4(5Z,8Z,11Z,14Z)/0:0], PE[20:4(8Z,11Z,14Z,17Z)/0:0], PE(22:0/0:0), and PE[22:6(4Z,7Z,10Z,13Z,16Z,19Z)/0:0] were positively associated with changes in *Streptomyces* (*R* > 0.6, *P* < 0.001). PE(21:0/0:0) was positively associated with changes in *Alistipes* and *Lachnospiraceae* UCG-001 (*R* > 0.6, *P* < 0.001). PE[20:1(11Z)/0:0] was positively correlated with *that of Alistipes* (*R* > 0.6, *P* < 0.001). However, the genera *Christensenellaceae* R-7 and *Allobaculum* were negatively correlated with PE[20:4(5Z,8Z,11Z,14Z)/0:0], PE[22:6(4Z,7Z,10Z,13Z,16Z,19Z)/0:0], PE(18:0/0:0), PE(21:0/0:0), LysoPE[0:0/22:6(4Z,7Z,10Z,13Z,16Z,19Z)], PE[20:4(8Z,11Z,14Z,17Z)/0:0], PE(22:0/0:0), and PE(20:1(11Z)/0:0) (*R* < −0.6, *P* < 0.001). The change in *Blautia* was negatively associated with PE(18:0/0:0) and PE(21:0/0:0) (*R* < −0.6, *P* < 0.001), and the change in *Ruminococcaceae* was negatively correlated with PE(21:0/0:0), PE(22:0/0:0), and PE[20:1(11Z)/0:0] (*R* < −0.6, *P* < 0.001). Moreover, the metabolites containing PE[22:6(4Z,7Z,10Z,13Z,16Z,19Z)/0:0] and PE[20:4(8Z,11Z,14Z,17Z)/0:0] were negatively associated with *Odoribacter*, *Parasutterella*, and *Lachnospiraceae* UCG-010 (*R* < −0.6, *P* < 0.001). PE(21:0/0:0) and LysoPE[0:0/22:6(4Z,7Z,10Z,13Z,16Z,19Z)] were correlated with changes in *Lachnospiraceae* UCG-010 (*R* < −0.6, *P* < 0.001). LysoPE(0:0/18:0) was negatively associated with *Allobaculum* (*R* < −0.6, *P* < 0.001), and LysoPE[0:0/22:6(4Z,7Z,10Z,13Z,16Z,19Z)] was negatively correlated with *Parasutterella* (*R* < −0.6, *P* < 0.001; Figure 6 and Supplementary Data 10).

Furthermore, 1-Arachidonoylglycerophosphoinositol was positively correlated with *Alistipes* and *Streptomyces* (*R* > 0.6, *P* < 0.001), but negatively correlated with *Christensenellaceae* R-7 *group*, *Ruminococcaceae* UCG-004, and *Allobaculum* (*R* < −0.6, *P* < 0.001). OHOHA-PS was positively associated with *Alistipes*, *Lachnospiraceae* UCG-001, and Streptomyces (*R* > 0.6, *P* < 0.001), whereas OHOHA-PS was negatively associated with Odoribacter, *Ruminococcaceae* UCG-005, *Christensenellaceae* R-7 group, *Ruminococcaceae* UCG-004, *Allobaculum*, and *Lachnospiraceae* UCG-010 (*R* < −0.6, *P* < 0.001). 1-(2-methoxy-eicosanyl)-sn-glycero-3-phosphoethanolamine was negatively correlated with changes in *the Christensenellaceae* R-7 *group* (*R* < −0.6, *P* < 0.001). Other low correlation results (R > −0.6 or R < 0.6) were shown in Figure 6 and Supplementary Data 10.

## Discussion

To test our hypothesis that the modification of plasma metabolites by ε-polylysine was related to the gut microbiota, we conducted breeding experiments on C57 mice. Weight loss was observed during the growth period, upon treatment with ε-polylysine, which may be attributable to the effects of lysine decomposition from ε-polylysine on animal metabolism rarely (28). Combined with ε-polylysine changes in the intestinal morphology of mice, which may be due to the influence of gut microbiota by ε-polylysine, or the regulation of glycerophospholipid metabolism to affect intestinal epithelial cells. Therefore, we investigated the regulatory effects of ε-polylysine on the intestinal microbiota and metabolites. ε-Polylysine changed the gut microbiota structure in this study, similar to a previous study (18, 19). The abundance of Firmicutes and Bacteroidetes were the predominant phyla in the gut microbiota during the experimental period, which was consistent with previous research results (29–31). Some symbiotic Actinobacterial species are probiotics, which can control bacterial diseases in livestock, poultry, and aquaculture by converting the feed stuffs into microbial biomass and fermentation end products for animal hosts (32). Actinobacteria may produce vitamin B12 (33) and some water-soluble vitamins (32). Phylum Actinobacteria played a role in gastrointestinal and systemic diseases and showed potential therapeutic effects (34). In this study, the abundance of the phylum Actinobacteria increased as the mice grew, while the abundance of Proteobacteria was increased at 4 and 6 weeks and decreased at 10 weeks, which resulted in the establishment of a mutualistic relationship with the host, and the abundance of Proteobacteria was increased in intestinal diseases and identified as a possible microbial signature of disease (35).

ε-Polylysine improved the relative abundance of beneficial bacteria and immunity in mice. At 4 weeks, ε-polylysine enhanced the abundance of probiotics such as *Faecalibaculum* (36), *Bifidobacterium* (37), and *Coriobacteriaceae* UCG-002 (38). The relative abundance of *Faecalibacterium*, *Butyricicoccus*, *and Lachnospiraceae* NK4B4 *group* were increased, but that of *Coriobacteriaceae* UCG-002, *Faecalibaculum*, *and Bifidobacterium* were decreased, indicating that the beneficial bacteria had complex regulation by ε-polylysine during the middle growing and adult periods of mice. Furthermore, ε-polylysine inhibited the abundance of potential biomarkers associated with diseases, including *Ruminococcus*, *Prevotella* 2 (39), *Prevotellaceae*, *Prevotellaceae* NK3B31 *group*, *Butyricimonas* (40), *Coprococcus* 2 (41), *Tyzzerella* 4 (42), *Escherichia-Shigella* (43), and *Subdoligranulum* (44). In addition, ε-polylysine decreased the abundance of the genus *Robinsoniella* separated from feces from premature infants (45), indicating that ε-polylysine may be a potential food additive for baby food. Moreover, ε-polylysine played an important role in the production of short-chain fatty acids (SCFAs), and high levels of SCFAs could inhibit inflammation, which was associated with an increased abundance of the genera *Ruminococcaceae* (46), *Collinsella* (47), *Blautia*, *Allobaculum*, *Alistipes*, *Prevotella*, *Bacterioides*, *and Butyricimonas* (48–50).

Importantly, ε-polylysine regulated lipid metabolism and reduced the likelihood of obesity and obesity-related diseases, which had important implications for animal health by regulating the relevant intestinal flora in C57 mice. During the post-weaning period of C57 mice, ε-polylysine restrained the abundance of the genera *Rikenellaceae* RC9 *gut group* and *Ruminococcaceae*, both of which were increased in obesity induced by a high-fat diet in rats, which indicated that ε-polylysine might regulate lipid metabolism and reduce the risk of obesity (50, 51). In addition, ε-polylysine downregulated the obesity-regulating genera *Collinsella*, *Negativibacillus*, and *Anaerotruncus* as well (52, 53). On the other hand, the abundance of *Parabacteroides* (54), *Odoribacter* (55), and *Alistipes* (56) were increased by ε-polylysine, a genus that played a positive regulatory role in lipid metabolism, and its content was negatively correlated with obesity and other diseases associated with fat metabolism. *Akkermansia muciniphila* can reduce the levels of relevant blood markers of inflammation and improve metabolic parameters (57, 58). In our study, the abundance of genus *Akkermansia* was increased by ε-polylysine, which showed a positive association with host metabolic health. The abundance of the genera *Akkermansia*, *Oscillibacter*, *Intestinimonas*, and *Alistipes* was significantly reduced in human fat (59), which contradicts the results of our study demonstrate. At 6 weeks, the middle growing period, the genera *Intestinimonas*, *Collinsella*, *Ruminococcaceae*, and *Lachnospiraceae* UCG-001 (60–62) were upregulated, which might be associated with weight loss from 5 to 7 weeks, and could also maintain the gut health of mice. In addition, ε-polylysine decreased the abundance of *Parabacteroides*, *Alistipes*, *Rikenellaceae* RC9 *gut group*, *Lactobacillus*, *Roseburia*, and *Marvinbryantia*. These results showed that lipid metabolism was regulated much more than in the post-weaning period, by ε-polylysine. In the adult period of mice, the genera *Allobaculum*, *Lachnospiraceae* UCG-010 (57), *Parasutterella* (63), *Ruminococcaceae*, *Odoribacter*, *Blautia*, *Holdemania* (64), and *Intestinimonas* were induced by ε-polylysine, which positively correlated with lipid metabolism. However, ε-polylysine reduced the genera *Lachnospiraceae* UCG-001, *Alistipes*, and *Parabacteroides*. The intestinal microbiota structure grew richer and more complex with age. Due to the regulation of ε-polylysine on gut microbiota, we evaluated further combinations of plasma metabolomics and correlation analyses.

PC and PE are the most abundant phospholipids in mammalian cell membranes, of which small alterations may have a large effect on parameters associated with metabolic syndrome, such as lipid profile, obesity, insulin resistance (65). Furthermore, the changes in PC and/or PE content in various tissues are associated with metabolic disorders disease like atherosclerosis, steatohepatitis, non-alcoholic fatty liver disease (66–68). Through statistical analysis, we found that levels of phospholipids: PCs, lysoPCs, PEs, LysoPEs, 1-Arachidonoylglycerophosphoinositol, 1-(2-methoxy-eicosanyl)-sn-glycero-3-phosphoethanolamine, and OHOHA-PS were decreased, whereas only those of PC[18:2(9Z,12Z)/22:5(4Z,7Z,10Z,13Z,16Z)] and PC[16:1(9Z)/20:3(8Z,11Z,14Z)] were increased, suggesting that phospholipid metabolism was affected by ε-polylysine, which corresponded to the upregulation of lipid metabolism predicted by PICRUSt of intestinal microbiota. It is well known that PCs are supplied by diet or synthesized in the liver *via* the Pemt pathway. PE converted to PC *via* sequential methylation reactions in the Pemt pathway (65). In this study, the level of PCs, PEs, and its decomposition products LysoPCs, LysoPEs of total glycerophospholipid were deduced compared ε-polylysine groups to control group, which was in accordance with changes in the Pemt pathway. Furthermore, changes in PC/PE radio were more concerned than changes in absolute concentrations of PC and PE. The cellular PC/PE molar ratio affects energy metabolism, and the importance of phospholipid metabolism in regulating lipid, lipoprotein, and whole-body energy metabolism (65). When he L*pcyt1a*^–/–^ mice fed with a high-fat diet, the PC levels and PC/PE radio decreased, and eventually L*Pcyt1a*^–/–^ mice developed non-alcoholic fatty liver disease (NAFLD). In L*Pcyt1a*^–/–^ and *Pemt*^–/–^ mouse models, the severity of NAFLD was inversely correlated with the hepatic PC/PE ratio (69, 70). Interesting, the PC and PE level decreased and the PC/PE radio was minimally affected by ε-polylysine in our study, which showed ε-polylysine as a food addictive had no negatively effects on the lipid metabolism.

The presence of gut microbes significantly elevated PC levels in the colon and significantly decreased PE levels in the ileum from GF and SPF mice (71). Moreover, fuzhuan brick tea ameliorated high-fat-diet-induced abnormal glycerophospholipid metabolism by increasing serum levels of PCs, LysoPCs, and PC/PE radio, which in a gut microbiota dependent manner (72). In the study of tumor, the gene *Alistipes* interfered glycerophospholipid metabolism, and it was negatively correlated with the level of PE and PC (73), which was contrary to our experimental results. The direct correlation study between gut microbes and metabolites in the glycerophospholipid metabolic pathway is rare, and indirect correlation studies have shown an important role in nutritional metabolic syndrome or health diseases (9, 74). This experiment required further research and verification on the correspond metabolism mechanism of glycerophospholipid with the microbiota directly.

In conclusion, the abundance of glycerophospholipid metabolites, including PC, PE, lysoPC, and lysoPE, was decreased by ε-polylysine. the genera *Alistipes*, *Lachnospiraceae* UCG-001, and *Streptomyces* were positively associated with PC, PE, LysoPC, LysoPE, 1-Arachidonoylglycerophosphoinositol and OHOHA-PS, but changes in *Blautia*, *Christensenellaceae* R-7 *group*, *Odoribacter*, *Allobaculum*, *Ruminococcaceae* UCG-004, *Ruminococcaceae* UCG-005, and *Lachnospiraceae* UCG-010 were negatively correlated with glycerophospholipid metabolites. The findings of our study demonstrated that ε-polylysine affected phospholipid metabolism, which was related to the effects of gut microbiota on C57 mice.

## Data Availability Statement

The original contributions presented in the study are publicly available. This data can be found here: https://www.ncbi.nlm.nih.gov/bioproject/, PRJNA752496. https://www.ebi.ac.uk/metabolights/, MTBLS4270.

## Ethics Statement

The animal study was reviewed and approved by Scientific Ethics Committee of Huazhong Agricultural University (approval number: HZAUMO-2019-059).

## Author Contributions

DW, XY, QD, and XZ designed the research. XZ, ZH, CX, and YN conducted the research. XZ and BX analyzed the data. XZ wrote the manuscript. All authors read and approved the final version of the manuscript.

## Conflict of Interest

The authors declare that the research was conducted in the absence of any commercial or financial relationships that could be construed as a potential conflict of interest.

## Publisher’s Note

All claims expressed in this article are solely those of the authors and do not necessarily represent those of their affiliated organizations, or those of the publisher, the editors and the reviewers. Any product that may be evaluated in this article, or claim that may be made by its manufacturer, is not guaranteed or endorsed by the publisher.
